# Organic Matter Enrichment and Reservoir Nanopore Characteristics of Marine Shales: A Case Study of the Permian Shales in the Kaijiang–Liangping Trough

**DOI:** 10.3390/nano15241870

**Published:** 2025-12-12

**Authors:** Xinrui Yang, Liangjun Xu, Huilin Li, Mingkai Zhang, Sirui Liu, Lu Xu, Dongxi Liu, Tong Xia, Jia Wang

**Affiliations:** 1 Chongqing Gas Field, PetroChina Southwest Oil and Gas Field Company, Chongqing 401120, China; 2 School of Petroleum Engineering, Chongqing University of Science and Technology, Chongqing 401331, China; 3 Chongqing Key Laboratory of Complex Oil and Gas Exploration and Development, Chongqing University of Science and Technology, Chongqing 401331, China

**Keywords:** paleoproductivity, organic matter, Wujiaping Formation, Dalong Formation, Kaijiang–Liangping Trough

## Abstract

To clarify the organic matter enrichment regularity of Permian shales in the Kaijiang–Liangping Trough, as well as the differential characteristics of their reservoir lithology, mineral assemblage, and nanopore structure—and thereby provide a geological basis for the exploration and development of Permian marine shales in the eastern Sichuan Basin—core samples from different depths of the Wujiaping Formation and Dalong Formation in Well DY-1H were analyzed using a series of micro–nano technical research methods, including whole-rock X-ray diffraction, major/trace element analysis, conventional porosity-permeability measurement, high-pressure mercury intrusion porosimetry, nitrogen adsorption, and field emission scanning electron microscopy. Research finds that the Dalong Formation shale contains Type I organic matter with high abundance, whereas the Wujiaping Formation shale is dominated by Type II_2_ organic matter. The Wujiaping Formation experienced stronger terrigenous input and higher weathering intensity, while the Dalong Formation was deposited under persistently anoxic conditions, in contrast to the frequent oxic–anoxic alternations in the Wujiaping Formation. Paleoproductivity indicators suggest higher productivity in the Dalong Formation than in the Wujiaping Formation. Mo/TOC ratios below 4.5 indicate deposition in a strongly restricted water body. Enrichment factors of multiple elements further support the enhanced paleoproductivity of the Dalong Formation. The Dalong Formation shale has higher contents of quartz and carbonate minerals, while the Wujiaping Formation shale has a higher content of clay minerals. The Wujiaping Formation shale is more developed with inorganic micropores, whereas the Dalong Formation shale is characterized by more developed organic nanopores. During the sedimentary period of the Dalong Formation shale, the paleoproductivity was high, the sedimentary waterbody had high reducibility and restriction, and the reservoir was well-developed with nanopores. The Dalong Formation is a more favorable interval for Permian shale gas exploration and development in the Kaijiang–Liangping Trough.

## 1. Introduction

In recent years, Sinopec has successively discovered one trillion-cubic-meter gas field and four 100-billion-cubic-meter gas fields in Fuling, Weirong, Qijiang, Yongchuan, and Hongxing [1]. PetroChina has built the largest domestic shale gas production base—the Southern Sichuan Shale Gas Field (the cumulative gas production had exceeded 80 billion cubic meters by the end of 2024). Since 2020, Sinopec has deployed and drilled Well HY1, Well Leye-1, and Well Mao-1 in the Hongxing area and Puguang Block; PetroChina has deployed and drilled Well DY-1H in the Kaijiang–Liangping area. These wells have successively obtained industrial gas flows ranging from 64,000 to 426,600 cubic meters per day in the Gufeng Member, Wujiaping Formation, and Dalong Formation of the Permian System. The Permian marine shale has shown promising shale gas exploration and development potential (with a geological resource volume of 17.81 × 10^12^ cubic meters and a recoverable resource volume of 2.67 × 10^12^ cubic meters), attracting the attention of many scholars at home and abroad [2,3].

The Permian organic-rich shales in the Upper Yangtze Region consist of three sets of shale sequences: the Gufeng Member of the Maokou Formation, the Wujiaping Formation (Longtan Formation), and the Dalong Formation (Changxing Formation). These shales are characterized by complex lateral facies changes [2]. The Kaijiang–Liangping Trough, a topographically low-lying area formed by intense rifting during the Late Permian, served as an ideal site for the deposition of high-quality Permian shales [4]. Previous studies have focused on the exploration potential [5], source rock conditions [6,7], and reservoir characteristics [8,9] of the Permian shales in the Kaijiang–Liangping Trough. However, research on the mechanisms of differential shale gas accumulation and reservoir differences remains relatively insufficient, which cannot meet the urgent needs of current efficient shale gas exploration and development. Therefore, this study conducts a comparative investigation into the mechanisms of organic matter enrichment and reservoir differences in the Wujiaping Formation and Dalong Formation under the Late Permian differential sedimentation model. It is expected to provide certain data support and geological suggestions for the optimization of favorable target areas in the exploration and development of Permian shale gas in China.

## 2. Regional Geological Setting

**Figure 1:**
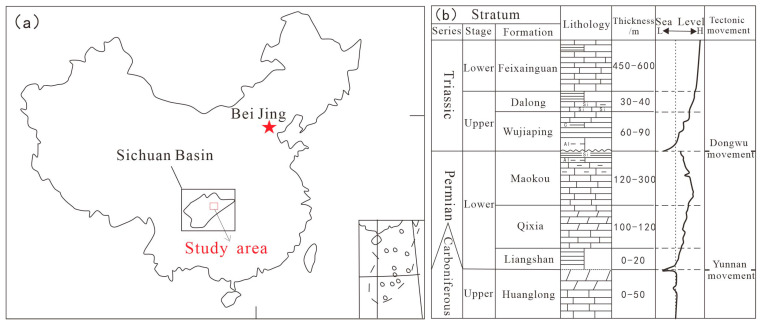
Regional location (**a**) and strata (**b**) of the Late Permian in the Kaijiang–Liangping Trough area.

During the Late Permian period, the entire Sichuan Basin was strongly influenced by the Dongwu Movement and the expansion of the Mianlue Ocean. In the southwest, the tectonic activity was dominated by the uplifting of the Emeishan Superplume. In the northeast, the oceanic crust of the Mianlue Ocean subducted beneath the Qinling Block [9], forming the NW-SE trending Kaijiang–Liangping Trough—a U-shaped trough opening towards Guangyuan–Wangcang and Liangping on both sides. The east–west differential tectonic-palaeogeographic framework of the Sichuan Basin resulted in significant contemporaneous heteropic sediments (sediments of the same age but different facies). In the early stage of the Late Permian, when the trough was initially rifted, sediments transited from continental to marine facies from west to east, sequentially developing the Xuanwei Formation sandstone, Longtan Formation coal seams/mudstone, and Wujiaping Formation shale (Figure 1a,b). In the late stage of the Late Permian, as sea level rose, organic-rich shale of the Dalong Formation was deposited in the trough, while carbonate platform and slope facies of the Changxing Formation were developed on both sides of the trough [10,11].

**Figure 2:**
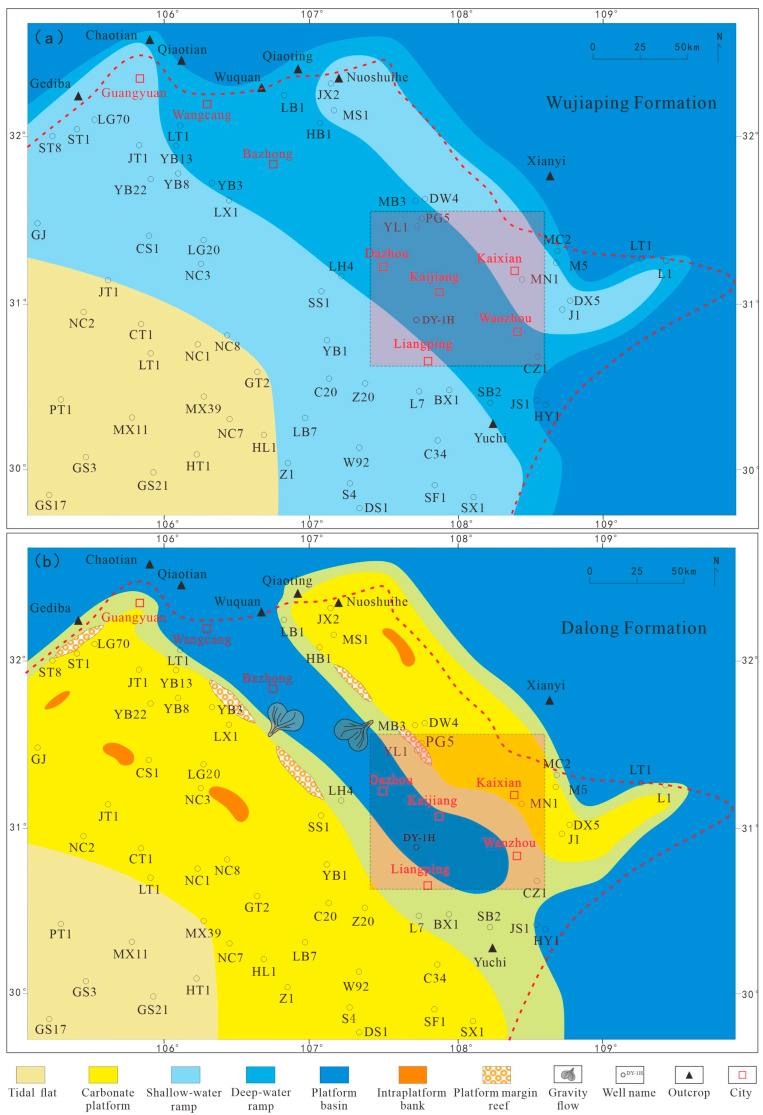
Sedimentary facies plan of the Permian Wujiaping Formation (**a**) and Dalong Formation (**b**) in the northeastern Sichuan Basin.

During the Late Permian, deep-water platform-basin facies sediments were developed in the Kaijiang–Liangping Trough, and significant changes also occurred in the sedimentary model [12]. During the Wujiaping Formation period, the sedimentary model was a carbonate ramp, and the trough generally exhibited the characteristics of being “gentle and wide” (Figure 2a). During the Dalong Formation deposition period, it gradually transitioned to a carbonate-rimmed platform sedimentary model, and the trough became steeper and narrower (Figure 2b). Compared with the deep-water shelf facies shales of the Longmaxi Formation in the Sichuan Basin, the Permian shales had a weaker sediment source during their deposition period and a higher carbonate mineral content (15–45%, with an average of 27.6%), which also resulted in the unique geological conditions of the Permian organic-rich shales.

## 3. Experimental Methods

To achieve the objective of studying the enrichment patterns of organic matter in shales and their differential characteristics, 38 Permian Wujiaping Formation and Dalong Small cylindrical samples of formation shale with a length of 10 cm and a diameter of 2.5 cm were collected from three high-yield shale gas DY-1H wells drilled by PetroChina Chongqing Gas Field (Chongqing, China) in the Kaijiang–Liangping Trough for whole-rock X-ray diffraction (XRD) analysis. Meanwhile, 38 of these powder samples were tested for major/trace element tests. A total of 36 shale samples were collected for reservoir porosity and permeability measurements and scanning electron microscopy (SEM) observations, and 3 samples for high-pressure mercury intrusion porosimetry (MIP) and nitrogen adsorption experiments. All the aforementioned experiments were conducted at the State Key Laboratory of Oil and Gas Reservoir Geology and Exploitation and the Chongqing Key Laboratory of Complex Oil and Gas Field Exploration and Development. The experimental procedures strictly adhered to the Petroleum and Natural Gas Industry Standards of the People’s Republic of China [13,14,15,16,17].

The experimental process was carried out as follows: First, no less than 100 g of shale particles were ground to below 200 mesh, and whole-rock XRD analysis was performed using a Rigaku SmartLab X-ray Diffractometer (Rigaku Corporation, Tokyo, Japan). During this experiment, the laboratory temperature was maintained at 20–25 °C with humidity ≤ 60%, and the experimental data were collected using MDI Jade software (MDI Jade 6.5.0.26, Materials Data, Inc., Livermore, CA, USA). After acidifying and digesting these samples with a 2–5% nitric acid (HNO_3_) solution, major and trace element analysis was completed with an Expec 7000 Inductively Coupled Plasma Mass Spectrometer (Focused Photonics Inc., FPI, Hangzhou, China). Subsequently, absolute ethanol was selected for the Soxhlet extraction of organic matter from the powder sample, and total organic carbon (TOC) determination was conducted using a Shimadzu TOC-L analyzer (Shimadzu Corporation, Kyoto, Japan). Next, shale particles were selected for field-emission scanning electron microscopy (FE-SEM) observations using a JEOL JSM-7800F microscope (JEOL Ltd., Tokyo, Japan). Shale samples ground to below 200 mesh were subjected to nitrogen adsorption experiments using a Quantachrome Quadrasorb SI instrument (Quantachrome Instruments, Boynton Beach, FL, USA). Finally, small cylindrical shale samples (2.5 cm in diameter, >5 cm in length) were drilled for conventional porosity and permeability experiments. Subsequently, these cylindrical samples were used for mercury intrusion experiments with a Micromeritics AutoPore IV 9510 Automatic Mercury Intrusion Porosimeter (Micromeritics Instrument Corporation, Norcross, GA, USA).

## 4. Results

### 4.1. Petrographic Description

**Figure 3:**
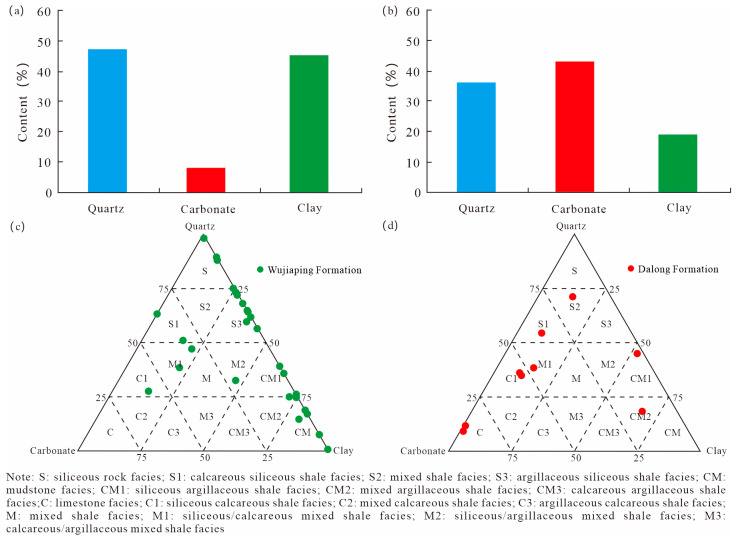
Triangular plot of mineral composition and lithology of Permian Shales in the Kaijiang–Liangping Trough. (**a**) Mineral composition of shales in the Dalong Formation; (**b**) mineral composition of shales in the Wujiaping Formation; (**c**) triangular diagram of shales in the Wujiaping Formation; (**d**) triangular diagram of shales in the Dalong Formation.

Whole-rock XRD data of 38 shale samples from Well DY-1H show that, under differential sedimentation, the shale samples from the Wujiaping Formation and Dalong Formation exhibit significant differences in mineral composition (Table 1). The Dalong Formation is characterized by high carbonate-mineral content and low clay-mineral content, with the highest carbonate mineral content (46.02%), followed by quartz (31.04%), and only 22.92% clay minerals, indicating that during the deposition period, the water depth was relatively deep (Figure 3a). In contrast, the Wujiaping Formation shale is characterized by high clay-mineral content and low quartz content; clay minerals account for the highest proportion (50.85%), with quartz at 39.52% and carbonate minerals at 9.63% (Figure 3b). Based on the mineral composition characteristics of the shales and in accordance with the three-end-member shale lithofacies-naming principle [12], the lithofacies of the Wujiaping Formation shales are mainly mixed shale facies, mudstone facies, and calcareous siliceous shale facies (Figure 3c). The Dalong Formation shales, on the other hand, are predominantly siliceous calcareous shale facies, mixed shale facies, calcareous siliceous shale facies, and mixed siliceous shale facies (Figure 3d).

### 4.2. Organic Geochemistry

Shales from both the Wujiaping Formation and Dalong Formation (Late Permian) are high-quality source rocks (Table 2). However, due to different sedimentary conditions and models, their organic geochemical parameters exhibit certain differences. The Dalong Formation shales have higher organic matter abundance than those of the Wujiaping Formation, with a Total Organic Carbon (TOC) content averaging as high as 2.79%. The organic matter type of the Dalong Formation is Type Ⅰ, and its parent material is mainly derived from plankton. In contrast, the Wujiaping Formation shales have an average TOC content of 1.32%, and their organic matter type is Type Ⅱ_2_. The hydrocarbon-generating parent material is dominated by phytoplankton. The vitrinite reflectance (Ro), an indicator of thermal maturity, is generally higher than 2.0%, and the average values all fall into the high-maturity thermal evolution stage.

### 4.3. Inorganic Geochemistry

The results of major/trace element analysis for 38 samples show that: the contents of major elements vary significantly, ranging from 0.0% to 76.2% with an average of 7.42%, and are dominated by SiO_2_, Fe_2_O_3_, and Al_2_O_3_; the contents of trace elements range from 0.0 ppm to 1436 ppm, and are mainly dominated by Zr, Cu, and Sr (Table 3 and Table 4).

### 4.4. Microscopic Reservoir Experimental Data

#### 4.4.1. Physical Properties

The conventional porosity and permeability experimental data of 24 shale samples show the following. For shales in the Wujiaping Formation: porosity ranges from 0.3% to 4.6%, with an average of 1.75%; permeability ranges from 0.012 mD to 0.03 mD, with an average of 0.023 mD. For shales in the Dalong Formation: porosity ranges from 0.2% to 2.9%, with an average of 1.38%; permeability ranges from 0.007 mD to 0.027 mD, with an average of 0.014 mD.

#### 4.4.2. Pore Types and Structures

**Figure 4:**
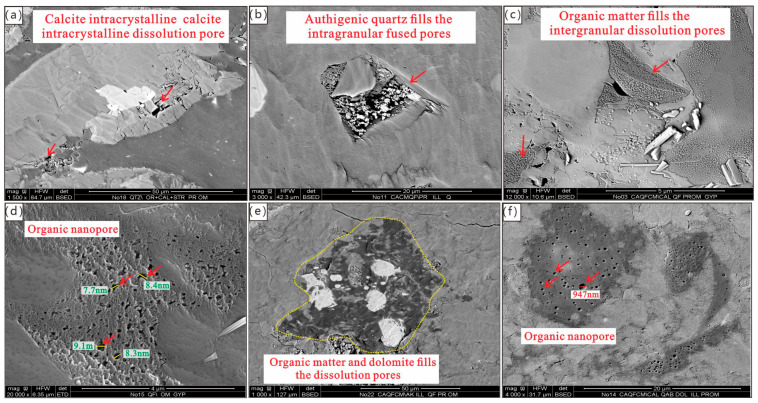
Nanopore microscopic characteristics of Permian shales in the Kaijiang–Liangping Trough. (**a**) Well DY-1H, 4333.12 m, Dalong Formation, calcite intracrystalline dissolution pore (as indicated by the red arrows in the figure), 3–5 μm; (**b**) Well DY-1H, 4336.17 m, Dalong Formation, authigenic quartz fills the intragranular fused pores (as indicated by the red arrows in the figure), 1–10 μm); (**c**) Well DY-1H, 4364.93 m, Wujiaping Formation, organic matter and gypsum fills the intergranular dissolution pores (as indicated by the red arrows in the figure); (**d**) Well DY-1H, 4336.78 m, Dalong Formation, organic nanopore (as indicated by the red arrows in the figure); (**e**) Well DY-1H, 4367.30 m, Wujiaping Formation, organic matter and dolomite fills the dissolution pores, the yellow dashed circle indicates the organic matter region; (**f**) Well DY-1H, 4337.80 m, Dalong Formation, organic nanopore (as indicated by the red arrows in the figure).

Observation via field-emission scanning electron microscopy (FE-SEM) reveals that, under strong compaction, the pore types of Permian shales mainly include intragranular dissolved pores of minerals (Figure 4a,b), intercrystalline pores of quartz (Figure 4c) and pyrite, and organic matter nanopores (Figure 4d–f). The pore diameter of intragranular dissolved pores in quartz, feldspar, and calcite typically ranges from 0.3 μm to 1.0 μm, and most of these pores are isolated. The diameter of pyrite intercrystalline pores usually ranges from 0.1 μm to 0.3 μm, and they are mostly fissure-like in shape. The diameter of organic matter nanopores generally ranges from 5 nm to 200 nm, often appearing as circular pore-like or long strip-like structures.

For the Permian shale, the mercury intrusion volume of high-pressure mercury intrusion ranges from 6.07% to 11.5%, the threshold pressure ranges from 0.18 MPa to 0.28 MPa, and the pore-throat radius ranges from 2.67 μm to 4.01 μm. The nitrogen adsorption experiment shows that the pore volume of the shale ranges from 1.43 cm^3^/g to 19.22 cm^3^/g, the BET specific surface area ranges from 0.0 m^2^/g to 0.03 m^2^/g, and the micropore diameter ranges from 0.0 nm to 1.95 nm.

## 5. Discussion

### 5.1. Mechanism and Pattern of Differential Enrichment of Organic Matter

By comprehensively utilizing the experimental analysis data of X-ray diffraction (XRD) and major and trace elements of Permian shale from the Well DY-1H, this study systematically reveals the differences in paleotectonic and paleosedimentary settings, paleoproductivity, redox conditions, and basin restriction degree during the sedimentary period of the Wujiaping Formation and Dalong Formation shales, as well as their influence mechanisms on the hydrocarbon generation potential of organic-rich shales.

#### 5.1.1. Paleoclimate

**Figure 5:**
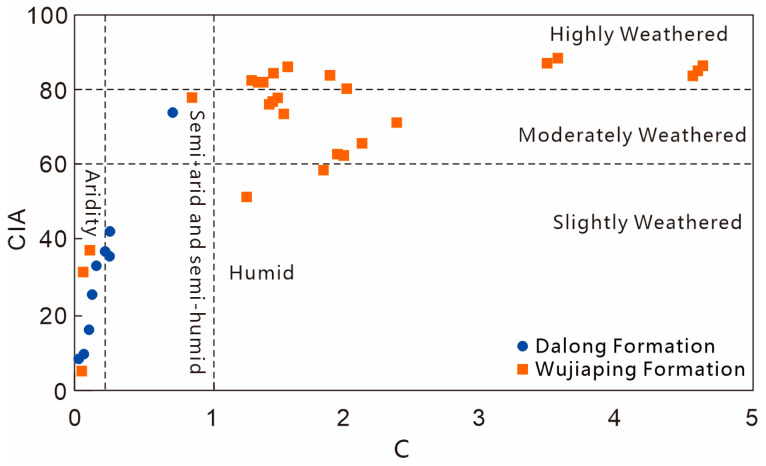
Paleoclimate discrimination chart of Late Permian shales in the Kaijiang–Liangping Trough.

The Chemical Index of Alteration (CIA, calculated as [Al_2_O_3_/(Al_2_O_3_ + CaO* + Na_2_O + K_2_O)] × 100) and the C-value (calculated as Σ(Fe + Mn + Cr + Ni + V + Co)/Σ(Ca + Mg + K + Na + Sr + Ba)) can reflect the humid or arid climate and weathering degree during the sedimentary period [18,19]. The calculation results and plotting of CIA values and C-values (Figure 5) show the following: for the Wujiaping Formation shales, the CIA values range from 4.86 to 90.61 with an average of 70.01, and the C-values range from 0.054 to 7.81 with an average of 2.40; for the Dalong Formation shales, the CIA values range from 8.24 to 73.9 with an average of 31.07, and the C-values range from 0.029 to 0.739 with an average of 0.23. The CIA values and C-values of the Wujiaping Formation are significantly higher than those of the Dalong Formation. The Wujiaping Formation was dominated by a humid climate and exhibited moderate to strong weathering characteristics, while the Dalong Formation was mainly characterized by arid and semi-arid to semi-humid climates, with relatively weak weathering degree.

#### 5.1.2. Redox Conditions

**Figure 6:**
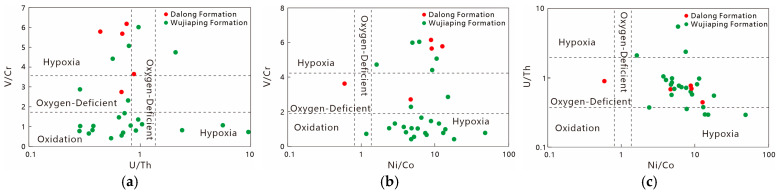
Redox discrimination chart of bimetallic elements in the Kaijiang–Liangping Trough during the Late Permian. (**a**) U/Th-V/Cr discrimination diagram; (**b**) Ni/Co-V/Cr discrimination diagram; (**c**) Ni/Co-U/Th discrimination diagram.

The redox degree of marine water is a key factor determining the differential enrichment of sensitive elements such as V, Cr, and U, as well as the preservation of organic matter, and has always been a research focus in environmental geochemistry [20,21]. Traditionally, higher values of V/Cr, U/Th, and Ni/Co indicate a higher degree of anoxia in water. Algeo found that the Corg/P ratio can reliably indicate redox conditions when algal organic matter is well-developed [22]; meanwhile, the redox properties of bottom water can be determined based on the Mo_EF_-U_EF_ diagram [23]. During the Late Permian, the Wujiaping Formation had an average V/Cr ratio of 1.88, while the Dalong Formation had an average V/Cr ratio of 4.78; the Wujiaping Formation had an average U/Th ratio of 1.80, while the Dalong Formation had an average U/Th ratio of 0.69; the Wujiaping Formation had an average Ni/Co ratio of 8.86, while the Dalong Formation had an average Ni/Co ratio of 7.23. Data on bimetallic element ratios show that the degree of anoxia of the Wujiaping Formation shale is lower than that of the Dalong Formation (Figure 6). Among them, the redox degree of the Wujiaping Formation varies greatly, indicating a transitional sedimentary environment.

**Figure 7:**
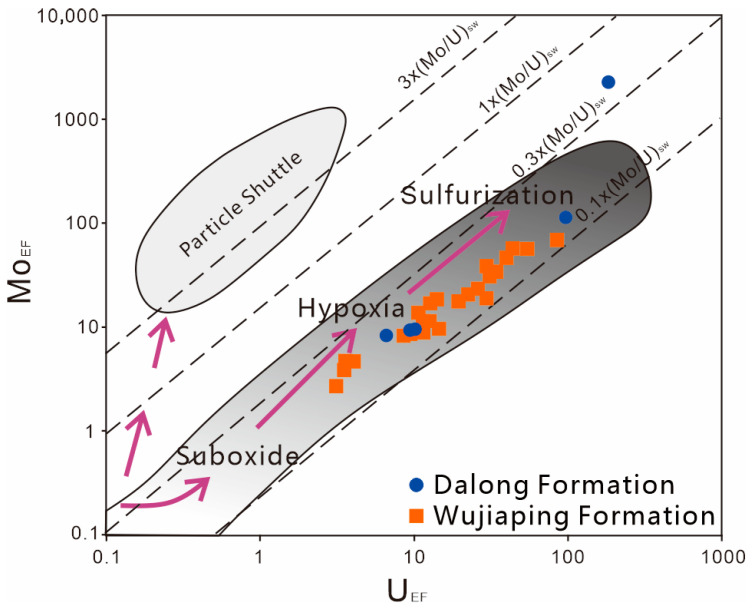
M_OEF_-U_EF_ diagram of Permian shale in the Kaijiang–Liangping Trough.

The C_org_/P ratio of the Dalong Formation ranges from 0.001 to 0.07 with an average of 0.03, while that of the Wujiaping Formation ranges from 0.001 to 0.42 with an average of 0.09. These data indicate that the reduction degree of the Wujiaping Formation is instead stronger than that of the Dalong Formation, which may be due to the fact that the Wujiaping Formation shale is not composed of algal organic matter [6], resulting in the false appearance of strong reduction. According to the M_OEF_-U_EF_ diagram (Figure 7), the M_OEF_-U_EF_ of the Late Permian shales shows a significant positive correlation and generally falls into the anoxic-sulfidic zone. This suggests that both the Wujiaping Formation and Dalong Formation shales exhibit strong reducibility, and the reducibility of the Dalong Formation shale is generally stronger than that of the Wujiaping Formation.

#### 5.1.3. The Degree of Water Body Limitation and Terrestrial Input

**Figure 8:**
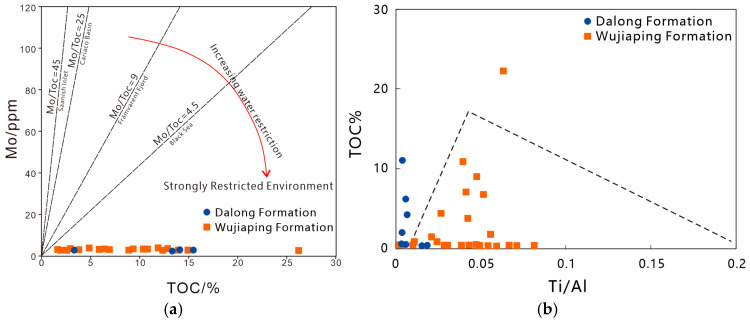
Discrimination diagram of water-body restriction and terrigenous input degree of Permian shale in the Kaijiang–Liangping Trough. (**a**) Mo-TOC relationship diagram; (**b**) (Ti/Al)-TOC relationship diagram.

The correlation between the content of Mo in shale and TOC (Total Organic Carbon) value can be used to determine the water restriction degree of sedimentary basins [24]. A lower Mo/TOC ratio indicates a higher water restriction degree of sedimentary water. Both the Dalong Formation and Wujiaping Formation shales fall into the strongly restricted environment (Figure 8a), with Mo/TOC ratios all less than 4.5, which is similar to the strongly restricted and anoxic-sulfidic water environment of the Black Sea.

Different amounts of terrigenous clastic input and sedimentation rates (SR) affect the enrichment degree of organic matter in the basin, and the Ti/Al ratio can indicate the amount of terrigenous clastic input and the magnitude of sedimentation rate. Plotting results of Permian shales (Figure 8b) show that the Ti/Al ratios of Dalong Formation shales are generally less than 0.045, and with the increase in sedimentation rate, terrigenous input increases and TOC values increase; the Ti/Al ratios of Wujiaping Formation shales are generally greater than 0.045, and with the increase in sedimentation rate, terrigenous input increases while TOC values decrease. This indicates that the Wujiaping Formation shale has stronger terrigenous input, and a high sedimentation rate dilutes the content of organic matter; in contrast, the Dalong Formation shale had weaker terrigenous input during the sedimentary period, and a high sedimentation rate facilitates the preservation of organic matter.

#### 5.1.4. Paleoproductivity

In recent years, total organic carbon (TOC), biogenic barium (Ba_bio_), and organic phosphorus (P_org_) have often been used to indicate the paleoproductivity of shales during their sedimentary period, but there are also many problems [25]. For example, only 0.1% to 10% of the organic matter in the surface seawater can be preserved after undergoing oxidative degradation; meanwhile, a large amount of terrigenous input will dilute the organic matter in seawater [6,26]. Additionally, phosphorus (P) and barium (Ba) elements undergo intense dissolution under strongly reducing conditions, leading to the underestimation of paleoproductivity. Therefore, scholars usually use the normalized values of enrichment factors of nutrient elements (Cu, Ni, and Zn) to indicate the paleoproductivity of seawater under reducing conditions [6,27].

The element enrichment factor (XEF) is calculated using the formula: X_EF_ = [(X/Al)sample/(X/Al)_PASS_], where (X/Al)PASS refers to the element-enrichment factor of the average Post-Archean Australian shale (PASS, a standard reference in geochemistry) element abundance. An XEF value greater than one indicates that the element is enriched relative to PASS, while a value less than one indicates depletion.

The calculation results of Ba_EF_ (barium enrichment factor), Cu_EF_ (copper enrichment factor), Zn_EF_ (zinc enrichment factor), and Ni_EF_ (nickel enrichment factor) for the Permian shales in the Kaijiang–Liangping Trough show (Table 5) that the Ba_EF_ and Cu_EF_ of the Dalong Formation shales are comparable to those of the Wujiaping Formation, but the Zn_EF_ and Ni_EF_ are significantly higher than those of the Wujiaping Formation. This proves that the seawater paleoproductivity during the sedimentary period of the Dalong Formation was higher than that of the Wujiaping Formation.

**Figure 9:**
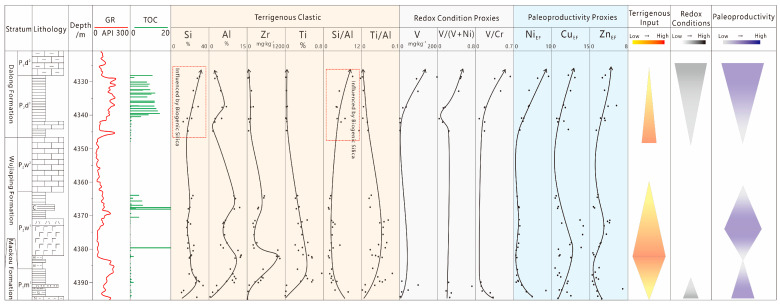
Vertical variation trend of paleoenvironment and paleoproductivity in the Late Permian of Well DY-1H.

Overall, the organic matter enrichment in Late Permian shales is comprehensively constrained by the paleosedimentary environment, terrigenous input, and paleoproductivity conditions (Figure 9). The Wujiaping Formation is dominated by semi-arid to humid climates, characterized by strong terrigenous input, high sedimentation rate, relatively low reducibility, and moderate to relatively high paleoproductivity. In contrast, the Dalong Formation is mainly dominated by arid climates: its terrigenous input is weakened (however, the presence of biogenic silica leads to the false appearance of strong terrigenous input in the Dalong Formation), with decreased sedimentation rate, increased reducibility, and high paleoproductivity. Consequently, the degree of organic matter enrichment in the Dalong Formation is naturally superior to that of the Wujiaping Formation.

#### 5.1.5. Pattern of Differential Enrichment of Organic Matter

**Figure 10:**
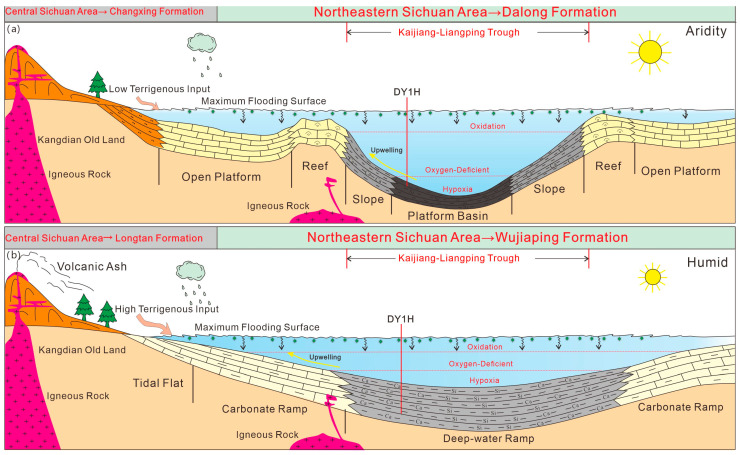
Differential enrichment model of the Permian Dalong Formation (**a**) and Wujiaping Formation (**b**) shale organic matter in the Kaijiang–Liangping Trough area.

Based on previous studies [3,4] and combined with the understanding of the paleosedimentary environment, paleoproductivity, and other factors, this study establishes a differential enrichment model of organic matter for the Wujiaping Formation and Dalong Formation in the Kaijiang–Liangping Trough area (Figure 10).

During the entire Late Permian, the provenance was mainly derived from the Kangdian Old Land. The Wujiaping Formation was deposited in carbonate ramp facies, with relatively strong provenance input and a predominantly humid environment. Deep-water ramp facies deposits in the trough developed argillaceous shales and calcareous shales, accompanied by extensive deposition of volcanic rocks and tuffs.

Later, with the intensification of rifting, during the Dalong Formation sedimentary period, carbonate-rimmed platforms were formed on both sides of the Tiandong-4 Well and Menxi-5 Well. Compared with the Wujiaping sedimentary period, the Dalong Formation had weaker provenance input, an arid climate, higher reducibility and water restriction degree, lower sedimentation rate, and stronger paleoproductivity. Within the trough, organic-rich siliceous shales and calcareous siliceous shales of deep-water platform-basin facies were formed.

### 5.2. Reservoir Differential Characteristics

Similar to conventional sandstone and carbonate reservoirs, the quality of shale reservoirs is also comprehensively constrained by early sedimentary mineral fabrics and late diagenetic evolution [28]. Meanwhile, the evolution of shale reservoirs is accompanied by complex thermal evolution of organic matter, which also renders the properties of shale diagenetic fluids more complex. The formation of inorganic pores and organic pores of different scales makes the pore structure of shale reservoirs diversified and micro-scale [29].

**Figure 11:**
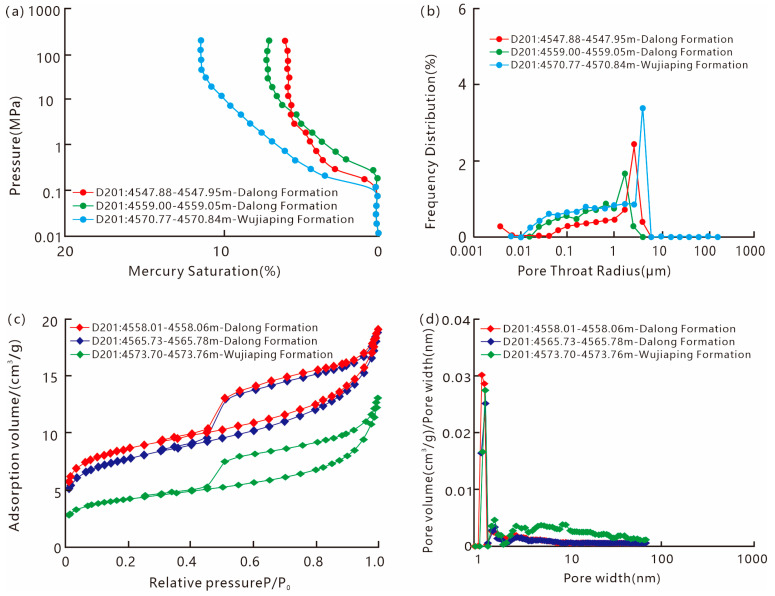
Pore structure characteristics of Permian shale reservoirs in the Kaijiang–Liangping Trough area. (**a**) Capillary pressure curve of high-pressure mercury injection; (**b**) high-pressure mercury injection pore size distribution; (**c**) capillary pressure curve of nitrogen adsorption; (**d**) nitrogen adsorption pore size distribution.

As mentioned earlier, under the differential sedimentary conditions of the Late Permian in the Kaijiang–Liangping Trough area, the Wujiaping Formation and Dalong Formation shales exhibit significant differences in mineral fabric and organic matter enrichment, which is bound to result in obvious differences between the two sets of shale reservoirs. From the experimental data, the high-pressure mercury intrusion curves of Permian shales all show a “high-steep” type (Figure 11a). The Wujiaping Formation shales have a higher mercury intrusion volume (Figure 11a) and more developed pores larger than 1 μm (Figure 11b), while the Dalong Formation shales have more developed 1~3 nm nanopores (Figure 11d). Overall, the Wujiaping Formation shales have more developed mesopores, while the Dalong Formation shales have more developed nano-scale pores, along with a larger specific surface area and stronger adsorption capacity (Figure 11c).

### 5.3. The Significance of Shale Gas Exploration

With the transition of the sedimentary model from a carbonate ramp to a rimmed platform during the Late Permian, the climate gradually became arid, terrigenous input gradually weakened, sedimentation rate gradually decreased, reducibility gradually increased, and both water restriction degree and paleoproductivity gradually enhanced. This consequently led to differences in mineral composition, organic matter content, and abundance of the organic-rich shales between the Wujiaping Formation and the Dalong Formation.

Overall, during the sedimentary period of Member 1 of the Dalong Formation, the water body was more restricted, the content of brittle minerals (quartz, carbonate minerals) was higher, and organic matter dominated by plankton was effectively preserved under strongly reducing conditions, with relatively more developed organic pores. From the perspective of shale gas exploration, the Dalong Formation shale is a more favorable development interval in the Kaijiang–Liangping Trough area and will become a key target horizon for subsequent shale gas exploration and development.

## 6. Conclusions

(1) The Wujiaping Formation in the Kaijiang–Liangping Trough area was deposited in a carbonate ramp setting. With the intensification of rifting, the Dalong Formation transitioned to a rimmed-platform sedimentary model, thereby governing the distribution of Permian shales, the patterns of organic matter enrichment, and the differential characteristics of reservoirs.
(2) During the Late Permian, the climate gradually transitioned from semi-arid and semi-humid to arid. Along with this transition, terrigenous input weakened, weathering gradually diminished, while reducibility, water restriction degree, and paleoproductivity gradually increased.
(3) In the study area, the Dalong Formation shale exhibits higher brittle mineral content, higher organic matter abundance, and more developed reservoir nanopores. It serves as a more favorable target horizon for subsequent shale gas exploration and development.

## Figures and Tables

**Table 1:** XRD analysis data of Permian Shales in the Kaijiang–Liangping Trough.

Serial Number	Well Number	Well Depth	Formation	Quartz (%)	Plagioclase (%)	Calcite (%)	Dolomite (%)	Pyrite (%)	Siderite (%)	Clay (%)
1	DY-1H	4328.21	P_2_*d*^1^	57.7	9.1	10	4.7	5.1	0.0	13.4
2	DY-1H	4328.80	P_2_*d*^1^	20.1	14.2	2.3	0.0	6.9	0.0	52
3	DY-1H	4332.50	P_2_*d*^1^	45.7	3.6	20	15.3	6.2	0.0	9.3
4	DY-1H	4337.20	P_2_*d*^1^	32.7	4	45	1.6	0.0	0.0	14.2
5	DY-1H	4337.45	P_2_*d*^1^	9.3	4.1	13.8	0.0	5.4	0.0	67.3
6	DY-1H	4340.75	P_2_*d*^1^	32.7	2.9	49.6	3.6	1.2	0.0	10
7	DY-1H	4340.78	P_2_*d*^1^	32.7	2.9	49.6	3.6	1.2	0.0	10
8	DY-1H	4341.80	P_2_*d*^1^	9.3	3.8	84.5	1.4	1	0.0	0.0
9	DY-1H	4344.40	P_2_*d*^1^	11.3	1	66	21.7	0.0	0.0	0.0
10	DY-1H	4363.85	P_2_*w*^2^	54.1	0.0	0.0	0.0	8.2	15.3	22.5
11	DY-1H	4364.70	P_2_*w*^2^	25.6	2.4	2.1	17.2	2.8	5.8	44
12	DY-1H	4367.30	P_2_*w*^2^	16.1	0.0	0.0	0.0	4.4	21.6	57.9
13	DY-1H	4367.79	P_2_*w*^2^	10.6	25.4	0.0	0.0	0.0	0.0	64
14	DY-1H	4371.23	P_2_*w*^2^	8.9	0.0	4.3	0.0	0.0	3	78.3
15	DY-1H	4371.76	P_2_*w*^2^	14.6	13.4	55.2	2.1	0.0	0.0	14.6
16	DY-1H	4372.27	P_2_*w*^2^	39.3	22.6	0.0	0.0	0.0	0.0	38.1
17	DY-1H	4373.22	P_2_*w*^1^	15.3	35.8	32.5	0.0	0.0	0.0	16.5
18	DY-1H	4375.56	P_2_*w*^1^	10	37.9	30.5	0.0	0.0	0.0	21.6
19	DY-1H	4377.94	P_2_*w*^1^	34.4	5	39.3	0.0	0.0	0.0	21.2
20	DY-1H	4378.60	P_2_*w*^1^	25.1	0.0	3.2	0.0	0.0	0.0	71.7
21	DY-1H	4379.46	P_2_*w*^1^	85.8	1.3	0.0	0.0	2.4	0.0	10.4
22	DY-1H	4380.24	P_2_*w*^1^	9.4	50.6	1.1	1.6	0.0	0.0	37.4
23	DY-1H	4381.54	P_2_*w*^1^	19	0.0	0.0	0.0	0.0	0.0	81
24	DY-1H	4381.80	P_2_*w*^1^	56.8	0.0	0.0	0.0	0.0	0.0	43.2
25	DY-1H	4382.22	P_2_*w*^1^	0.0	0.0	0.0	0.0	0.0	0.0	77.2
26	DY-1H	4382.45	P_2_*w*^1^	7.6	0.0	0.0	0.0	0.0	0.0	92.4
27	DY-1H	4385.77	P_2_*w*^1^	75.3	0.0	0.0	0.0	0.0	0.0	24.7
28	DY-1H	4387.18	P_2_*w*^1^	0.0	0.0	0.0	0.0	0.0	0.0	100
29	DY-1H	4388.01	P_2_*w*^1^	17.5	0.0	0.0	0.0	0.0	0.0	82.5
30	DY-1H	4388.65	P_2_*w*^1^	25.3	0.0	0.0	0.0	0.0	0.0	74.7
31	DY-1H	4389.12	P_2_*w*^1^	51.4	0.0	0.0	0.0	13.5	0.0	35.1
32	DY-1H	4389.47	P_2_*w*^1^	35.9	21	0.0	0.0	7.5	0.0	35.7
33	DY-1H	4389.75	P_2_*w*^1^	19.8	14.9	0.0	0.0	4.6	0.0	60.7
34	DY-1H	4390.62	P_2_*w*^1^	65.3	0.0	0.0	0.0	3.1	0.0	31.6
35	DY-1H	4392.04	P_2_*w*^1^	86.5	0.0	0.0	0.0	1.9	0.0	11.6
36	DY-1H	4392.61	P_2_*w*^1^	63.2	0.0	0.0	0.0	9.1	0.0	27.7
37	DY-1H	4394.00	P_2_*w*^1^	94.4	0.0	0.0	0.0	5.6	0.0	0.0
38	DY-1H	4395.15	P_2_*w*^1^	62.8	0.0	36.5	0.0	0.7	0.0	0.0

**Table 2:** Table of parameters for organic geochemical characteristics of Permian shales in the Kaijiang–Liangping Trough [6].

Formation	TOC (%)	Ro (%)	S_1_ + S_2_ (mg/g)	Tmax (°C)	δ^13^C_org_	Types of Organic Matter
Dalong Formation	0.17~10.98/2.79	1.67~2.42/2.14	0.01~0.68/0.16	317~597/543	−28.7~−22.7/−25.9	Ⅰ
Wujiaping Formation	0.05~22.28/1.32	1.95~3.18/2.75	0.01~0.87/0.07	449~562/514	−27.6~−21.1/−23.9	Ⅱ_2_

Note: Ro = 0.3364 + 0.6569Rb.

**Table 3:** Data table of major elements in Permian shales of the Kaijiang–Liangping Trough.

Serial Number	Well Number	Well Depth	Formation	Fe_2_O_3_ (%)	Mn (%)	Ti (%)	CaO (%)	K_2_O (%)	S (%)	P (%)	SiO_2_ (%)	Al_2_O_3_ (%)	MgO (%)
1	DY-1H	4328.21	P_2_*d*^1^	2.494	0.009	0.013	7.150	1.328	0.133	0.008	64.598	6.120	0.128
2	DY-1H	4328.80	P_2_*d*^1^	2.096	0.008	0.009	12.405	0.919	0.087	0.016	59.619	4.433	0.063
3	DY-1H	4332.50	P_2_*d*^1^	4.554	0.026	0.029	13.916	2.084	0.256	0.007	50.670	8.745	0.118
4	DY-1H	4337.20	P_2_*d*^1^	3.936	0.099	0.046	1.687	2.941	0.117	0.006	61.374	13.033	0.154
5	DY-1H	4337.45	P_2_*d*^1^	2.984	0.714	0.031	14.959	2.184	0.090	0.013	49.178	9.873	0.255
6	DY-1H	4340.75	P_2_*d*^1^	3.272	0.131	0.031	17.283	2.123	0.039	0.019	46.843	9.436	0.255
7	DY-1H	4340.78	P_2_*d*^1^	2.899	0.301	0.009	23.855	1.031	0.025	0.012	36.799	4.700	1.611
8	DY-1H	4341.80	P_2_*d*^1^	3.027	0.128	0.038	41.826	0.673	0.012	0.012	27.660	4.329	0.127
9	DY-1H	4344.40	P_2_*d*^1^	0.900	0.063	0.029	32.835	0.494	0.014	0.014	38.470	2.983	0.219
10	DY-1H	4363.85	P_2_*w*^2^	10.298	0.156	0.261	3.356	2.698	0.085	0.033	49.689	18.546	0.339
11	DY-1H	4364.70	P_2_*w*^2^	11.836	0.167	0.216	2.062	2.867	0.286	0.011	46.546	19.726	0.279
12	DY-1H	4367.30	P_2_*w*^2^	14.406	0.082	0.295	6.021	2.274	0.122	0.011	42.717	22.839	0.172
13	DY-1H	4367.79	P_2_*w*^2^	28.364	0.261	0.237	1.016	1.861	0.058	0.013	39.402	18.891	0.480
14	DY-1H	4371.23	P_2_*w*^2^	17.189	0.099	0.462	5.039	0.070	0.006	0.024	36.707	12.263	1.242
15	DY-1H	4371.76	P_2_*w*^2^	15.850	0.105	0.353	5.763	0.131	0.010	0.021	39.219	11.059	0.953
16	DY-1H	4372.27	P_2_*w*^2^	15.485	0.089	0.273	6.054	0.342	0.012	0.019	44.111	10.478	0.916
17	DY-1H	4373.22	P_2_*w*^1^	18.214	0.124	0.334	7.769	0.081	0.007	0.021	36.217	10.899	1.438
18	DY-1H	4375.56	P_2_*w*^1^	17.343	0.162	0.354	6.893	0.121	0.008	0.021	37.426	11.628	1.350
19	DY-1H	4377.94	P_2_*w*^1^	22.416	0.039	0.476	1.164	0.285	0.016	0.007	38.565	13.465	1.414
20	DY-1H	4378.60	P_2_*w*^1^	12.733	0.026	0.212	1.587	0.270	0.005	0.016	51.853	8.707	0.878
21	DY-1H	4379.46	P_2_*w*^1^	15.272	0.161	0.361	10.071	0.099	0.006	0.019	38.147	10.708	0.904
22	DY-1H	4380.24	P_2_*w*^1^	7.864	0.060	0.279	1.860	2.863	0.016	0.006	49.931	21.652	0.289
23	DY-1H	4381.54	P_2_*w*^1^	16.195	0.089	0.375	0.316	3.307	0.006	0.005	46.205	24.080	0.255
24	DY-1H	4381.80	P_2_*w*^1^	10.644	0.018	0.491	1.520	3.088	0.005	0.006	44.736	23.826	0.176
25	DY-1H	4382.22	P_2_*w*^1^	14.714	0.023	0.559	0.388	2.782	0.007	0.007	42.796	23.441	0.252
26	DY-1H	4382.45	P_2_*w*^1^	20.073	0.041	0.564	1.330	2.147	0.007	0.008	41.449	19.454	0.370
27	DY-1H	4385.77	P_2_*w*^1^	18.958	0.151	0.259	0.880	2.032	0.005	0.013	47.911	15.942	0.381
28	DY-1H	4387.18	P_2_*w*^1^	19.480	0.042	0.768	1.150	2.024	0.005	0.029	41.416	17.814	0.574
29	DY-1H	4388.01	P_2_*w*^1^	6.993	0.009	0.442	0.940	3.293	0.072	0.027	56.525	19.055	0.227
30	DY-1H	4388.65	P_2_*w*^1^	6.980	0.031	0.468	0.405	2.855	0.182	0.007	57.124	20.043	0.187
31	DY-1H	4389.12	P_2_*w*^1^	6.191	0.011	0.580	0.337	2.948	0.084	0.006	61.518	17.486	0.206
32	DY-1H	4389.47	P_2_*w*^1^	4.007	0.009	0.329	1.264	2.509	0.078	0.007	64.245	13.138	0.177
33	DY-1H	4389.75	P_2_*w*^1^	7.462	0.067	0.557	0.478	3.780	0.082	0.004	56.059	18.850	0.246
34	DY-1H	4390.62	P_2_*w*^1^	2.963	0.000	0.031	0.532	1.158	0.104	0.004	72.401	5.692	0.063
35	DY-1H	4392.04	P_2_*w*^1^	2.203	0.024	0.011	28.319	1.032	0.117	1.891	28.834	6.813	0.087
36	DY-1H	4392.61	P_2_*w*^1^	3.465	0.101	0.016	49.003	0.384	0.142	0.011	24.171	2.702	0.101
37	DY-1H	4394.00	P_2_*w*^1^	0.900	0.015	0.000	16.606	0.121	0.032	0.006	58.460	0.834	0.115
38	DY-1H	4395.15	P_2_*w*^1^	0.421	0.005	0.002	2.798	0.308	0.014	0.004	76.218	1.835	0.000

**Table 4:** Data table of trace elements in Permian shales from the Kaijiang–Liangping Trough.

Serial Number	Well Number	Well Depth	Formation	Ba (mg/kg)	Zr (mg/kg)	Sr (mg/kg)	Zn (mg/kg)	Cu (mg/kg)	Ni (mg/kg)	Cr (mg/kg)	V (mg/kg)	Co (mg/kg)	La (mg/kg)	Sc (mg/kg)	Th (mg/kg)	Mo (mg/kg)	U (mg/kg)
1	DY-1H	4328.21	P_2_*d*^1^	4.2	71.0	619.0	84.3	62.9	199.3	312.3	199.0	13.7	6.4	4.3	5.1	3.1	4.5
2	DY-1H	4328.80	P_2_*d*^1^	70.1	33.0	1161.0	48.4	65.0	97.9	257.1	90.7	26.3	9.8	6.2	6.3	2.5	4.3
3	DY-1H	4332.50	P_2_*d*^1^	45.2	140.0	1436.0	112.2	103.2	121.0	212.3	97.6	25.3	22.5	6.5	2.6	2.5	4.6
4	DY-1H	4337.20	P_2_*d*^1^	6.9	198.0	123.0	315.7	92.5	182.1	211.7	35.9	16.7	4.6	6.9	11.4	2.3	3.7
5	DY-1H	4337.45	P_2_*d*^1^	70.5	118.0	738.0	160.9	78.7	131.4	112.7	18.5	22.8	18.3	5.7	10.1	2.7	3.9
6	DY-1H	4340.75	P_2_*d*^1^	6.8	112.0	652.0	85.2	24.7	62.5	35.2	2.4	14.7	25.4	14.5	6.7	3.3	4.3
7	DY-1H	4340.78	P_2_*d*^1^	3.8	51.0	792.0	61.0	19.7	41.1	22.3	0.0	19.8	30.9	5.7	1.9	1.9	3.8
8	DY-1H	4341.80	P_2_*d*^1^	12.6	86.0	953.0	58.1	31.5	34.4	25.1	4.2	22.4	26.1	13.8	2.3	1.7	3.9
9	DY-1H	4344.40	P_2_*d*^1^	26.5	52.0	666.0	15.2	30.9	14.8	28.6	4.7	13.6	22.8	12.6	5.8	3.6	4.5
10	DY-1H	4363.85	P_2_*w*^2^	287.9	545.0	328.0	122.9	41.3	44.8	105.0	0.0	18.5	31.4	13.5	10.8	2.1	3.1
11	DY-1H	4364.70	P_2_*w*^2^	303.0	530.0	248.0	118.6	31.2	43.3	91.8	0.0	16.1	20.7	9.7	6.5	2.3	4.2
12	DY-1H	4367.30	P_2_*w*^2^	136.8	358.0	318.0	56.7	79.8	87.9	230.7	0.0	24.9	3.1	4.9	2.1	2.4	4.4
13	DY-1H	4367.79	P_2_*w*^2^	111.6	342.0	240.0	64.1	60.6	76.6	143.0	0.0	14.8	8.0	4.1	4.4	2.6	3.3
14	DY-1H	4371.23	P_2_*w*^2^	63.9	251.0	116.0	219.0	222.8	49.8	53.2	0.0	14.9	19.5	9.5	11.3	2.4	4.2
15	DY-1H	4371.76	P_2_*w*^2^	30.8	200.0	129.0	174.1	410.7	45.8	47.2	0.0	24.0	27.8	8.7	5.5	2.8	5.3
16	DY-1H	4372.27	P_2_*w*^2^	169.3	193.0	156.0	184.1	61.3	43.5	37.4	0.0	26.5	57.5	13.9	13.1	3.1	3.8
17	DY-1H	4373.22	P_2_*w*^1^	13.3	217.0	190.0	136.7	220.6	53.8	49.8	0.0	30.7	4.7	4.2	0.3	2.5	4.1
18	DY-1H	4375.56	P_2_*w*^1^	24.3	221.0	132.0	170.4	230.6	48.5	49.4	0.0	30.7	12.4	6.1	0.7	2.1	3.9
19	DY-1H	4377.94	P_2_*w*^1^	10.3	314.0	109.0	126.3	166.1	49.9	59.9	0.0	18.3	41.8	8.4	17.9	2.4	6.2
20	DY-1H	4378.60	P_2_*w*^1^	22.8	196.0	96.0	76.6	98.5	32.2	22.6	0.0	22.6	27.3	6.6	4.3	2.6	4.5
21	DY-1H	4379.46	P_2_*w*^1^	0.9	222.0	196.0	82.4	200.9	34.0	48.9	0.0	18.8	18.2	6.5	3.7	2.7	3.4
22	DY-1H	4380.24	P_2_*w*^1^	82.3	970.0	293.0	39.9	81.0	30.5	38.8	0.0	22.2	43.2	4.0	1.5	2.9	3.6
23	DY-1H	4381.54	P_2_*w*^1^	161.0	925.0	249.0	62.5	113.7	28.4	29.7	0.0	12.5	82.8	14.6	12.2	2.5	3.5
24	DY-1H	4381.80	P_2_*w*^1^	6.2	1001.0	274.0	43.8	114.7	26.4	31.4	0.0	15.2	11.5	5.2	7.9	3.2	5.4
25	DY-1H	4382.22	P_2_*w*^1^	14.1	930.0	275.0	59.6	98.8	30.7	32.0	0.0	14.4	15.6	6.4	6.1	3.3	4.3
26	DY-1H	4382.45	P_2_*w*^1^	76.0	1033.0	279.0	78.2	231.5	38.3	35.5	0.0	25.8	32.2	8.2	5.0	2.3	4.1
27	DY-1H	4385.77	P_2_*w*^1^	4.6	506.0	218.0	123.4	24.9	39.5	84.5	0.0	14.1	20.2	7.0	6.5	2.5	3.6
28	DY-1H	4387.18	P_2_*w*^1^	142.3	500.0	265.0	71.4	76.8	35.4	95.8	0.0	133.5	143.8	25.4	12.9	2.6	4.9
29	DY-1H	4388.01	P_2_*w*^1^	7.1	463.0	328.0	23.0	61.7	45.9	22.7	0.0	61.5	4.0	2.7	0.6	2.8	5.8
30	DY-1H	4388.65	P_2_*w*^1^	48.2	505.0	245.0	98.0	105.1	44.0	28.9	0.0	17.2	38.0	9.5	10.5	2.3	5.7
31	DY-1H	4389.12	P_2_*w*^1^	73.4	431.0	232.0	124.2	178.8	53.7	95.3	0.0	21.9	10.2	7.7	4.5	2.2	3.5
32	DY-1H	4389.47	P_2_*w*^1^	161.7	256.0	244.0	17.9	124.1	43.4	136.1	13.3	8.6	19.4	6.4	6.7	2.5	5.3
33	DY-1H	4389.75	P_2_*w*^1^	57.5	356.0	218.0	29.2	73.1	28.8	125.3	0.0	16.5	21.6	9.2	5.9	2.6	4.6
34	DY-1H	4390.62	P_2_*w*^1^	78.7	68.0	76.0	15.0	57.1	37.7	932.1	98.4	25.1	16.1	8.8	5.4	2.9	3.9
35	DY-1H	4392.04	P_2_*w*^1^	133.1	34.0	737.0	205.1	40.7	205.1	374.7	45.9	20.1	18.5	7.9	10.2	3.2	5.8
36	DY-1H	4392.61	P_2_*w*^1^	153.1	32.0	426.0	15.3	25.8	30.2	45.4	12.3	13.1	14.4	3.9	5.6	3.1	5.5
37	DY-1H	4394.00	P_2_*w*^1^	7.1	8.0	182.0	5.4	10.0	14.1	15.2	4.9	22.6	16.7	7.3	11.3	2.4	3.7
38	DY-1H	4395.15	P_2_*w*^1^	9.5	11.0	59.0	18.7	6.2	4.4	2.9	0.0	19.2	24.9	6.8	13.4	2.9	3.9

**Table 5:** Parameter table of element-enrichment coefficients of Permian Shales in the Kaijiang–Liangping Trough.

Formation	Ba_EF_	Cu_EF_	Zn_EF_	Ni_EF_	Number of Samples
Dalong	(0.21–0.48/0.40)	(1.54–8.63/4.99)	(1.13–5.35/2.91)	(1.34–8.78/3.71)	(9)
Wujiaping	(0.027–1.66/0.52)	(0.92–21.87/5.33)	(0.27–6.65/1.66)	(0.30–8.11/1.26)	(29)

## Data Availability

The original contributions presented in this study are included in the article. Further inquiries can be directed to the corresponding author.
